# Depression and fatigue in active IBD from a microbiome perspective—a Bayesian approach to faecal metagenomics

**DOI:** 10.1186/s12916-022-02550-7

**Published:** 2022-10-17

**Authors:** Anne Kerstin Thomann, Torsten Wüstenberg, Jakob Wirbel, Laura-Louise Knoedler, Philipp Arthur Thomann, Georg Zeller, Matthias Philip Ebert, Stefanie Lis, Wolfgang Reindl

**Affiliations:** 1 Department of Medicine II, University Medical Centre Mannheim, Medical Faculty Mannheim, Heidelberg University, Mannheim, Germany; 2 Core Facility for Neuroscience of Self-Regulation (CNSR), Field of Focus 4 (FoF4), Heidelberg University, Heidelberg, Germany; 3 European Molecular Biology Laboratory, Structural and Computational Biology Unit, Heidelberg, Germany; 4 Center for Mental Health, Odenwald District Healthcare Centre, Erbach, Germany; 5 Clinical Cooperation Unit Healthy Metabolism, Centre of Preventive Medicine and Digital Health, Medical Faculty Mannheim, Heidelberg University, Mannheim, Germany; 6 Department of Clinical Psychology, Central Institute of Mental Health, Medical Faculty Mannheim, Heidelberg University, Mannheim, Germany; 7 Department of Psychosomatic Medicine and Psychotherapy, Central Institute of Mental Health, Medical Faculty Mannheim, Heidelberg University, Mannheim, Germany

**Keywords:** Inflammatory bowel diseases, Microbiome, Depression, Fatigue, Brain-gut axis

## Abstract

**Background:**

Extraintestinal symptoms are common in inflammatory bowel diseases (IBD) and include depression and fatigue. These are highly prevalent especially in active disease, potentially due to inflammation-mediated changes in the microbiota-gut-brain axis. The aim of this study was to investigate the associations between structural and functional microbiota characteristics and severity of fatigue and depressive symptoms in patients with active IBD.

**Methods:**

We included clinical data of 62 prospectively enrolled patients with IBD in an active disease state. Patients supplied stool samples and completed the questionnaires regarding depression and fatigue symptoms. Based on taxonomic and functional metagenomic profiles of faecal gut microbiota, we used Bayesian statistics to investigate the associative networks and triangle motifs between bacterial genera, functional modules and symptom severity of self-reported fatigue and depression.

**Results:**

Associations with moderate to strong evidence were found for 3 genera (*Odoribacter*, *Anaerotruncus* and *Alistipes*) and 3 functional modules (pectin, glycosaminoglycan and central carbohydrate metabolism) with regard to depression and for 4 genera (*Intestinimonas*, *Anaerotruncus*, *Eubacterium* and Clostridiales g.i.s) and 2 functional modules implicating amino acid and central carbohydrate metabolism with regard to fatigue.

**Conclusions:**

This study provides the first evidence of association triplets between microbiota composition, function and extraintestinal symptoms in active IBD. Depression and fatigue were associated with lower abundances of short-chain fatty acid producers and distinct pathways implicating glycan, carbohydrate and amino acid metabolism. Our results suggest that microbiota-directed therapeutic approaches may reduce fatigue and depression in IBD and should be investigated in future research.

**Supplementary Information:**

The online version contains supplementary material available at 10.1186/s12916-022-02550-7.

## Background

The inflammatory bowel diseases (IBD) Crohn’s disease (CD) and ulcerative colitis (UC) are characterised by abdominal symptoms such as pain and diarrhoea, caused by intestinal inflammation. However, the quality of life of many persons with IBD is often considerably reduced by extraintestinal symptoms like fatigue or comorbid depression, which are highly prevalent especially in active IBD [1, 2]. In the last decade, studies of microbiota-gut-brain interactions have helped to gain a better understanding of functional gastrointestinal disorders and their relationship with mental health [3], and these interactions are now also increasingly addressed in IBD [4, 5].

There is ample data demonstrating that the gut microbiota is influenced by the onset of IBD and does not revert to its normal state during the quiescent phases of the disease [6]. Global microbial parameters like diversity and abundances of single taxa are associated with IBD incidence, prevalence or outcomes [7–10]. However, causal relationships or treatments targeting microbial dysbiosis have not been reliably established as the molecular mechanisms underlying these associations are still unclear [11].

The microbiome interacts closely with the intestinal immune system and has the ability to influence brain function while at the same time being subject to modulation by the central nervous system [12, 13]. Over recent years, it has also been associated with a growing number of mental disorders [14]. With regard to depression [15], alpha- and beta diversity have been shown to differ between patients and controls [16, 17]. Furthermore, a recent large study showed a depletion of specific taxa, such as *Dialister* and *Coprococcus*, to be associated with depressive symptoms [18]. Advances in sequencing techniques and analytic methods have allowed further insights into functional parameters of the microbiome such as the faecal metabolome in depression [19], linking depressive symptoms to alterations in the microbial amino acid metabolism.

Associations between the microbiome and fatigue have been analysed in patients with myalgic encephalitis/chronic fatigue syndrome [20, 21], multiple sclerosis and cancer [22] and non-alcoholic steatohepatitis [23] with heterogeneous fatigue-related results regarding diversity and specific taxa, which is not surprising considering that different disease entities are independently associated with microbiota changes.

In IBD, research on associations between the gut microbiome and extraintestinal symptoms is scarce. One descriptive study applied 16S rRNA gene sequencing on intestinal biopsies and associated depressive symptoms with the abundance of *Bifidobacterium* and *Desulfovibrio* in remitted patients with CD and UC, respectively [24]. Another recent study also using amplicon sequencing methods described the structural changes in the faecal microbiome (such as lower operational taxonomic unit (OTU) richness) in a small group of patients with IBD and depressive symptoms compared to others without depressive symptoms [25]. With regard to fatigue, one important recent study [26] specifically investigated the faecal metagenome of IBD patients with and without fatigue to determine associations between this common, but underinvestigated symptom and the microbiome. The authors reported a reduced bacterial diversity and reduced abundance of butyrate-producing bacteria, including *Ruminococcus*, *Faecalibacterium* and *Roseburia*, to be associated with fatigue. Although patients in this study were in clinical and endoscopic remission, more than half suffered from fatigue. In active disease, the prevalence of fatigue is reported to increase up to 80% [1], indicating a contribution of inflammatory processes in the development of fatigue. As mentioned above, depression is also much more prevalent in active disease, which may at least partly relate to similar mechanisms [2]. As participants of all mentioned IBD-related studies addressing the microbiome and its association with depression or fatigue were in remission, but both are more prevalent in active disease, it is necessary to obtain information about possible biomarkers of extraintestinal symptoms also in the presence of inflammation, i.e. during active disease.

In microbiome research, the microbial metabolome has attracted increasing scientific interest in recent years [27, 28]. As certain metabolic products are associated with a specific function, the approach of connecting changes in microbial abundance with changes in metabolic activity and psychometry can result in a more functional understanding of this interaction.

In this study, we obtained stool samples of patients with active disease and aimed to investigate associations between taxonomic and metabolic characteristics of the faecal microbiome and fatigue and depression.

## Methods

### Sample collection

Patients with active IBD were recruited from the IBD outpatient unit at Department of Medicine II, Medical Faculty Mannheim, Heidelberg University, between January 2018 and October 2019. The study procedures were approved by the ethics committee of the Medical Faculty Mannheim, Heidelberg University (2014-633N-MA), and conducted in accordance with the Declaration of Helsinki. All participants gave written informed consent after a thorough explanation of the study protocol. Active disease was defined by the presence of intestinal inflammation, determined by endoscopy, MRI, sonography and/or repeatedly elevated faecal calprotectin levels (> 250 mg/kg). Patients with recent use of antibiotics (< 4 weeks before recruitment) were excluded from the study. At the time of recruitment, we collected blood and stool samples as well as information on clinical disease activity (Harvey Bradshaw Index or partial Mayo Score, respectively), and patients completed the questionnaires regarding depression and fatigue.

Participants were asked to collect fresh stool samples at home and immediately freeze them at − 20 °C in their home freezer in 10-ml Falcon tubes (buffer-free) and a cooling bag and to bring them to the outpatient unit at the time of induction of the new therapy, where they were collected and immediately transferred to a − 80 °C freezer to prevent thawing.

### Questionnaires

Depressive symptoms were measured with Hospital Anxiety and Depression Scale, Subscale for Depression (HADS-D [29]). This self-reported screening instrument is widely used in different mental and somatic disorders and has been validated in patients with IBD [30]. It contains 14 items, 7 each for depression and anxiety symptoms, and scores range from 0 to 21 points for each subscale, with higher scores indicating higher depression and anxiety. A score of 10 points or higher on each subscale indicates at least moderate symptom load.

Fatigue was measured with Wurzburg Fatigue Inventory Multiple Sclerosis (WEIMuS [31]). This 17-item self-report instrument was developed for patients with multiple sclerosis but was previously used and well-accepted in patients with IBD [32]. WEIMuS scores reach from 0 to 68 points, with higher scores indicating more fatigue. The cut-off for relevant fatigue symptom load is generally considered to be at 32 points.

### DNA extraction

DNA extraction of the stool samples was conducted at the research lab at the Department of Medicine II, Medical Faculty Mannheim, Heidelberg University. We retrieved 500-μg aliquots of each stool sample. DNA was purified with the QIAamp Fast DNA Stool Mini Kit (Qiagen Cat. No. 51604) according to the protocol “Isolation of DNA from Stool for Pathogen Detection” (handbook pages 23–25, version March 2014). After the lysis of cells, proteins were digested and degraded using proteinase K. The DNA was then purified by silica membrane columns. The eluted DNA had an average concentration of 40 ng/μl.

### Metagenomics

Metagenomics was carried out by Novogene Europe Co. Ltd. (Cambridge, UK). Experimental procedures of metagenomic sequencing consisted of sample quality assessment, library construction and sequencing. The quality of DNA samples was tested as follows: (1) DNA degradation degree and potential contamination were monitored on 1% agarose gels, (2) DNA purity (OD260/OD280, OD260/OD230) was checked using the NanoPhotometer® spectrophotometer (IMPLEN, CA, USA) and (3) DNA concentration was measured using Qubit® dsDNA Assay Kit in Qubit®2.0 Fluorometer (LifeTechnologies, CA, USA). OD values between 1.8 and 2.0 and DNA contents above 1 μg are used to construct the library. A total amount of 1 μg DNA per sample was used as input material for the DNA sample preparations. Sequencing libraries were generated using NEBNext® Ultra™ DNA Library Prep Kit for Illumina (NEB, USA) following the manufacturer’s recommendations. Index codes were added to attribute sequences to each sample. Briefly, the DNA samples were fragmented by sonication to a size of 350 bp, then DNA fragments were end-polished, A-tailed and ligated with the full-length adaptor for Illumina sequencing with further polymerase chain reaction (PCR) amplification. At last, PCR products were purified (AMPure XP system), and libraries were analysed for size distribution by Agilent2100 Bioanalyzer and quantified using real-time PCR. The clustering of the index-coded samples was performed on a cBot Cluster Generation System according to the manufacturer’s instructions. After cluster generation, the library preparations were sequenced on an Illumina HiSeq platform with 150-bp reads with a mean of 23.9 million reads (SD = 3.4 million) per sample.

### Taxonomic and functional annotation

Samples were processed with a pipeline implemented in *NGLess* [33]: low-quality reads were filtered out using MOCAT2 [34], and reads mapping to the human reference genome (version hg38.p10) were removed. Taxonomic profiles were then generated with the mOTUs2 software, version 2.5.1 [35] and combined at different taxonomic levels. Functional profiling of metagenomes was conducted by mapping filtered reads against the integrated gene catalogue of the human gut microbiome [36] and then aggregating the counts for different orthologous groups (KOs) of the Kyoto Encyclopedia of Genes and Genomes (KEGG) database [37]. Lastly, the abundance of functional modules was calculated as the sum of reads across KO members according to the KEGG definitions. Since the manually curated KEGG module database does not include modules for the production of short-chain fatty acids (SCFAs), we extended the definitions by additionally including four metabolic pathways for the production of SCFAs as described in Vital et al. [38].

### Data filtering and adjustment

Further preprocessing steps and analyses were performed using the statistical software R (version 3.6.3, https://www.r-project.org/) and the *phyloseq* package for R (McMurdie & Holmes, 2013).

Taxonomic annotations were pooled at the genus level, resulting in 210 genera. Functional annotations were preprocessed at the module level, resulting in 235 modules. For five patients, the number of annotated genera or modules was below 50 (~ 20%). These patients were removed from further analyses. For the remaining 57 patients with annotations for 210 genera and 235 modules, data were prepared for analyses using a three-step approach:

1. *Prevalence filtering*: To focus on more prevalent taxa and to avoid overfitting during adjustment for nuisance variables, we removed further 151 genera and 26 modules that were prevalent in less than 15 patients. Although this leads to an unusual reduction in taxa, this aggressive filtering was necessary to allow correction for 10 confounding variables (see step 3) by means of a multiple regression model with at least 3 degrees of freedom. According to Cao and colleagues, this step very likely does not affect the integrity of data [39].
2. *CLR transformation*: Due to the compositional nature of microbiome data sets [40], the abundances were centred log ratio (CLR) transformed.
3. *Adjustment for nuisance variables*: To account for the known influence of host and environmental variables on the human microbiome [41–43], we adjusted CLR-scaled relative abundances for age, sex, inflammation (C-reactive protein, CRP) and medication (steroids, mesalamine, immunosuppressants, hormonal contraceptives [44], antidepressants, proton pump inhibitors). To this end, a multiple regression model containing 10 nuisance variables as predictors was fitted genus-wise to the data. All further analysis steps were carried out using the residuals of this regression.

### Analyses of the associations between psychopathology, alpha diversity and clinical inflammation markers

Associations between depression and fatigue and alpha diversity quantified by the Shannon diversity index [45] as well as current inflammatory status (quantified by CRP) were analysed by bivariate correlation analysis.

### Network construction and topological analysis

Graph-theoretical approaches are becoming increasingly important in biomedical science [46, 47]. Specifically, graph theoretical methods are very well suited to model complex relations within biological networks and to discover important mechanisms by means of the topological properties of these networks. To our knowledge, associative patterns among taxonomic and metabolic characteristics of individual microbiomes and individual expression of depressive symptomatology and fatigue have not been investigated before. Here, we base our analysis on a recent study [48], which proposed a framework of correlation and association analyses in the microbiome and integrative multiomics studies that offers an analytic approach to this constellation. According to the authors, a triple association between the composition of the microbiome could be associated with host factors as well as environmental factors or covariates including clinical or experimental conditions. In the present study, we identified triple associations of taxa and metabolic modules associated with each other and with the expression of depressive symptoms or fatigue and searched for topological network features, called triangular motifs, within the common network created by means of our outcome variables.

To this end, we computed the joint network for our three outcome parameters. The 270 nodes of this network were formed by the taxa and KEGG modules annotated for our faecal samples and the two psychometric scores. Connections between nodes were computed by means of Bayesian correlation analyses using the CLR-scaled abundances or symptom severities. Computations were conducted with the R package *BayesFactor* (https://CRAN.R-project.org/package=BayesFactor) using a weakly informed Jeffreys-Zellner-Siow (JZS) prior with an r scale of 0.354. Evidence for H1 was assessed by means of Bayes factors (BF)10 [49]. Subsequently, BFs were linearised using the common logarithm (Log_10_). According to Kass and Raftery [49], substantial evidence for H1 is indicated by Log_10_(BF10) ≥ 0.5; the evidence is strong in the case of Log_10_(BF10) ≥ 1.0 and decisive if Log_10_(BF10) ≥ 2.0 [49]. A connection between two nodes was considered for network construction in case of at least moderate evidence for H1.

Subsequently, we investigated the topology of this joint network regarding the patterns that linked taxonomy, metabolism and psychopathology. Those patterns, also called triangular motifs are formed by taxonomical-metabolic, taxonomical-psychopathological and metabolic-psychopathological associations and could be a sign of metabolism-mediated interactions between the gut microbiome and depression and fatigue severity. To assess the directions of associations within these motifs, we computed Spearman correlation coefficients. All findings are reported, displayed and discussed according to these criteria.

**Fig. 1:**
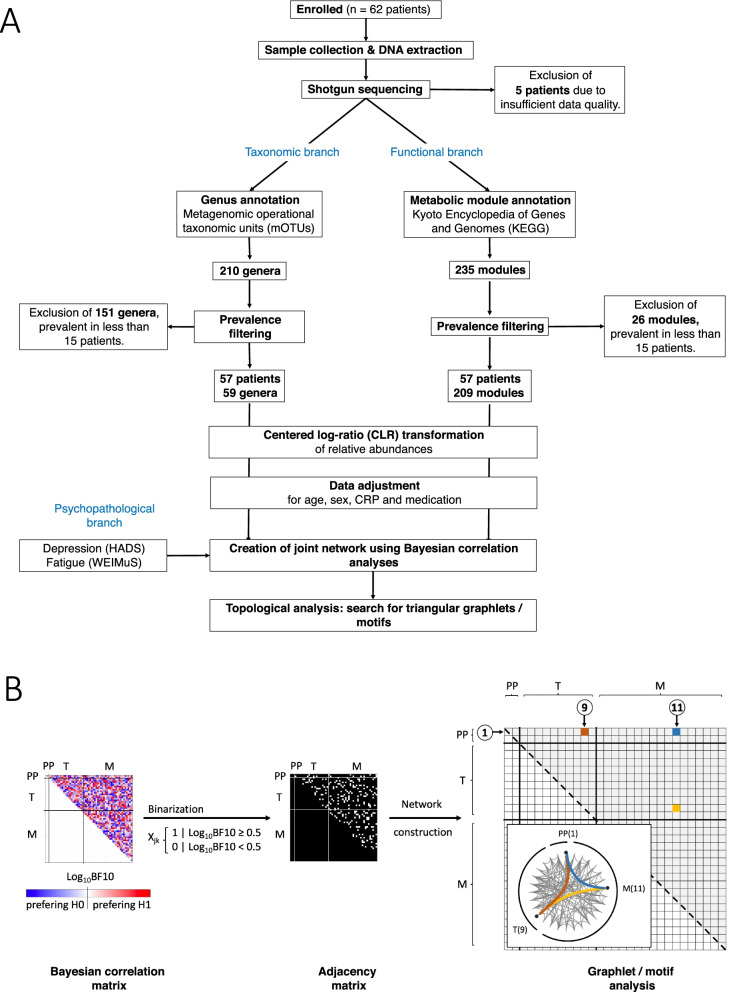
Data processing pipeline. **A** Displayed are the processing steps applied for data preprocessing (cleaning, prevalence filtering, CLR transformation and adjustment for nuisance variables) and analysis. **B** Graphical display of the main steps for network construction and motif analysis. From left to right: (1-left) Computation of the joint Bayesian correlation matrix. Because for undirected graphs, the correlation matrix is symmetrical, and only their upper part is considered in further steps. Colour codes the strength of evidence for a certain connection with white to red preferring H1 and white to blue preferring H0. Sections associated with psychopathology (PP, depression and fatigue), taxonomical (T, genera) and metabolic (M, KEGG modules) abundances are separated by thin black lines. (2-middle) To binarise this matrix, a threshold of Log_10_BF10 ≥ 0.5 was applied. For the remaining matrix elements or node connections, H1 is at least 3 times more likely than H0. The resulting binary adjacency matrix was used to construct the association network. (3-right) Exemplary representation of one triangular motif of interest, that is composed of interconnected nodes of all three modalities

The whole data processing pipeline is displayed in Fig. 1.

## Results

### Clinical characteristics of the study sample

We recruited 84 patients with active IBD. Twenty-two patients were excluded (*n* = 21 due to missing stool samples, *n* = 1 patient withdrew consent), leaving data of 62 patients available for analysis.

Demographic and clinical information is summarised in Table 1. Half of the patients reported fatigue scores above the cut-off value of 32 points and 18 patients reported depression scores indicating at least moderate symptom severity (HADS depression subscale of 10 points or higher).

**Table 1:** Demographic and clinical information of the study sample

Demographic and clinical information	Results
Age, years, mean (SD)	40 (16)
Sex, *n* (female/male)	36/26
Diagnosis (CD/UC)	51/11
HBI in patients with CD, median (range)	9.4 (6.8)
Partial Mayo Score in patients with UC, mean (SD)	5.6 (2.5)
CRP in mg/l, mean (SD)	21.2 (24.8)
Faecal calprotectin in μg/g, mean (SD) (*n* = 53)	365 (282)
Fatigue (WEIMuS) score, mean (SD)	31.5 (14.7)
WEIMuS ≥ 32P., *n* (%)	31 (50%)
Depression (HADS-D) Score, mean (SD)	6.5 (4.5)
HADS-D ≥ 10P., *n* (%)	18 (29%)
Current antidepressant use	4 (6%)
Current steroid use, *n* (%)	21 (34%)
Current immunomodulatory therapy, *n* (%)	13 (21%)
Of which biological therapy, *n*	10
Of which TNF-alpha inhibitors, *n*	3
Of which vedolizumab, *n*	5
Of which ustekinumab, *n*	2
Refractory disease course (> 3 prior systemic therapies), *n* (%)	22 (35%)
Prior bowel resection, *n* (%)	24 (38%)
Prior biological therapy, *n* (%)	31 (50%)

*CD* Crohn’s disease, *CRP* C-reactive protein, *HBI* Harvey-Bradshaw Index, *HADS* Hospital Anxiety and Depression Scale, *SD* standard deviation, *UC* ulcerative colitis, *WEIMuS* Wurzburg Fatigue Inventory Multiple Sclerosis

The cohort showed elevated markers of systemic and luminal inflammation with mean CRP levels of 21.2 mg/l and mean faecal calprotectin levels (available of *n* = 53 patients) of 365 μg/g. Neither biomarker was associated with fatigue (CRP: Log_10_(BF10) − 0.74; fCal: Log_10_(BF10) − 0.676) nor depression severity (CRP: Log_10_(BF10) − 0.76; fCal: Log_10_(BF10) − 0.580), Fig. 3.

Twenty-one patients were treated with systemic steroids at the time of study inclusion, and 10 patients with biologicals. Thirty-one patients had previously been treated with biologicals, and 31 were naïve. Twenty-two patients had a refractory disease course, i.e. had previously failed 3 or more systemic anti-inflammatory therapies. Twenty-four patients had undergone prior IBD-related surgery. Patients with refractory disease course, current steroid use and prior biological therapy had numerically lower depression scores, but neither of the current or previous medication and neither of the reported disease characteristics were significantly associated with depression or fatigue severity (Additional file 1: Table S1).

### Taxonomic sample characteristics

The number of annotated genera per patient ranged from 14 to 91 (median = 41) with genera mainly from the Firmicutes and Bacteroidetes phyla. Cumulative relative abundances of these annotated genera ranged from 0.334 to 0.995 (median = 0.942).

**Fig. 2:**
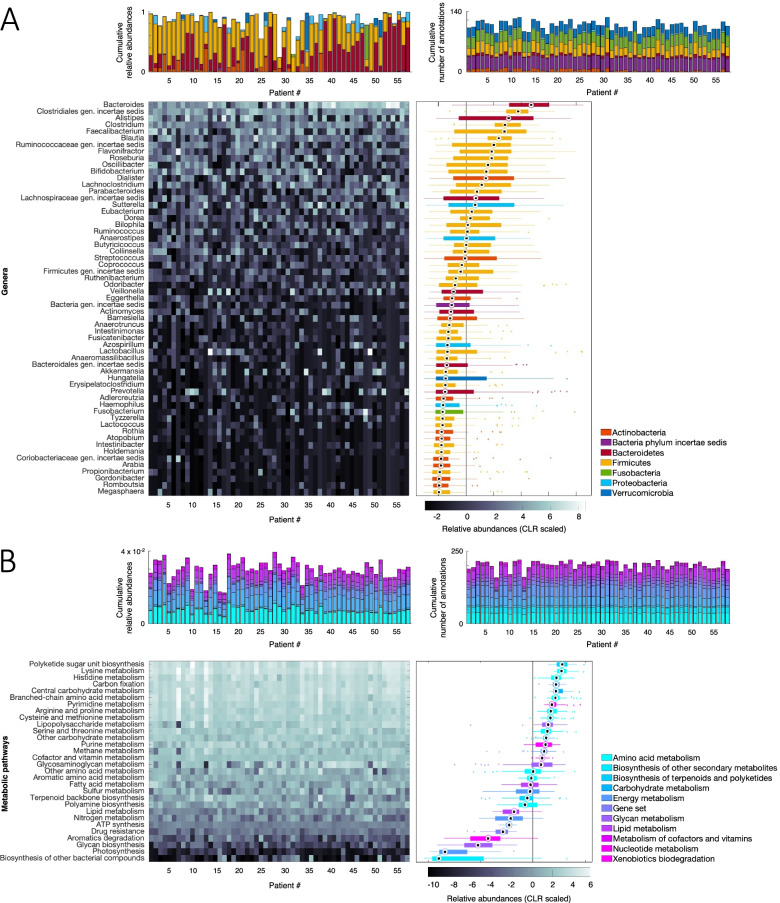
Taxonomic and metabolic sample characteristics. Stacked bar graphs show relative abundances of annotated genera (**A**, upper part) and KEGG modules (**B**, upper part). Heatmaps of relative abundances are summarised by boxplots for genera (**A**, bottom part) and KEGG modules (**B**, bottom part)

After the prevalence check, 59 genera annotated in at least 15 patients remained. The number of annotated genera per patient ranged from 14 to 53 (median 34), with Firmicutes and Bacteroidetes as the dominant phyla. Cumulative relative abundances over all phyla ranged from 0.2427 to 0.994 (median = 0.903). The highest abundances were found for Firmicutes (median = 0.412) and Bacteroidetes (median = 0.310). The final taxonomic annotation characteristics at the genus and phylum levels are shown in Fig. 2A.

### Metabolic sample characteristics

For the initially annotated 235 metabolic modules defined according to the KEGG database (https://www.genome.jp/kegg/module.html), the number of annotations per patient ranged from 139 to 209 (median = 188) with cumulative relative abundances over all modules ranging from 0.017 to 0.0.40 (median = 0.030).

After the prevalence check, 209 modules annotated in at least 15 patients each remained. The number of annotated modules per patient ranged from 139 to 201 (median 185). Mainly represented were modules belonging to the amino acid and carbohydrate pathways. Cumulative relative abundances over all modules ranged from 0.017 to 0.994 (median = 0.903). Highest abundances were found for the amino acid (median = 0.0074) and carbohydrate (median = 0.0071) pathways. The final metabolic annotation characteristics at the KEGG pathway level are shown in Fig. 2B.

### Associations with inflammatory activity

**Fig. 3:**
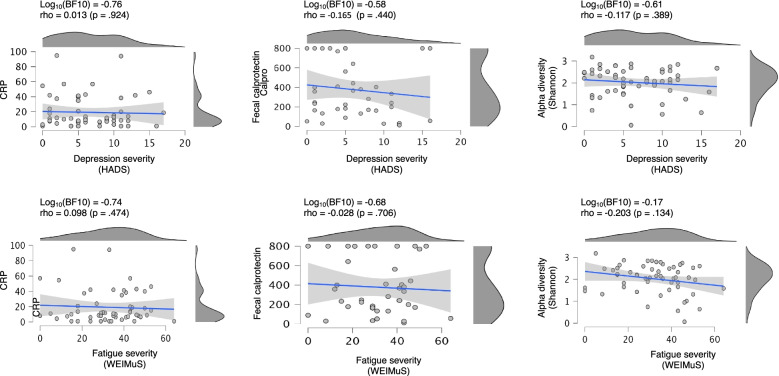
Correlations between biomarkers and depression/fatigue. Left column: scatter plots of CRP levels versus severity of depression and fatigue respectively. Middle column: scatter plots of faecal calprotectin levels versus severity of depression and fatigue, respectively. Right column: scatter plots of gut microbial alpha diversity (Shannon index) and depression and fatigue severity. Additionally shown are linear regression models (blue lines) and the 90% confidence interval for the model slope. Bayesian factors and Spearman correlation coefficients are given on top

*Systemic inflammatory activity*: Neither depression (Log_10_BF10 = − 0.76) nor fatigue (Log_10_BF10 = − 0.74) were associated with CRP levels (Fig. 3 (left)).

*Luminal inflammatory activity*: Neither depression (Log_10_BF10 = − 0.58) nor fatigue (Log_10_BF10 = − 0.68) were associated with faecal calprotectin levels (Fig. 3 (middle)).

### Association with microbiome diversity

*Shannon alpha diversity*: Neither depression (Log_10_BF10 = − 0.61) nor fatigue (Log_10_BF10 = − 0.17) were associated with alpha diversity (Fig. 3 (right)).

### Triangular network motifs consisting of nodes of taxonomical, functional and psychopathological data

*Depression*: Six association triples were found containing the *Odoribacter*, *Alistipes* and *Anaerotruncus* genera and carbohydrate and glycan metabolism associated modules. Most importantly, *Odoribacter* as well as glycan metabolism modules were each involved in three association triplets and showed very strong evidence for a positive reciprocal association (Log_10_(BF10) = 2.611, see the “Methods” section for definition). *Odoribacter* abundance was strongly negatively associated with depression severity (Log_10_(BF10) = 1.956) whereas the evidence for an (negative) association between glycan metabolism and depression was only moderate (Log_10_(BF10) = 0.530). In all triplets, below-average taxonomical and functional abundances were related to increased depression severity.

*Fatigue*: Four association triplets were found containing *Intestinimonas*, *Eubacterium*, *Anaerotruncus* and *Clostridiales g.i.s*. and carbohydrate and amino acid metabolism-associated modules. Evidence for associations was mostly moderate, with the exception of strong evidence supporting a negative association between *Clostridiales g.i.s*. or *Eubacterium* and carbohydrate metabolism (Log_10_(BF10) = 2.900 and 1.082, respectively). In all triplets, the above-average taxonomical and below-average functional abundances were related to increased fatigue severity.

**Fig. 4:**
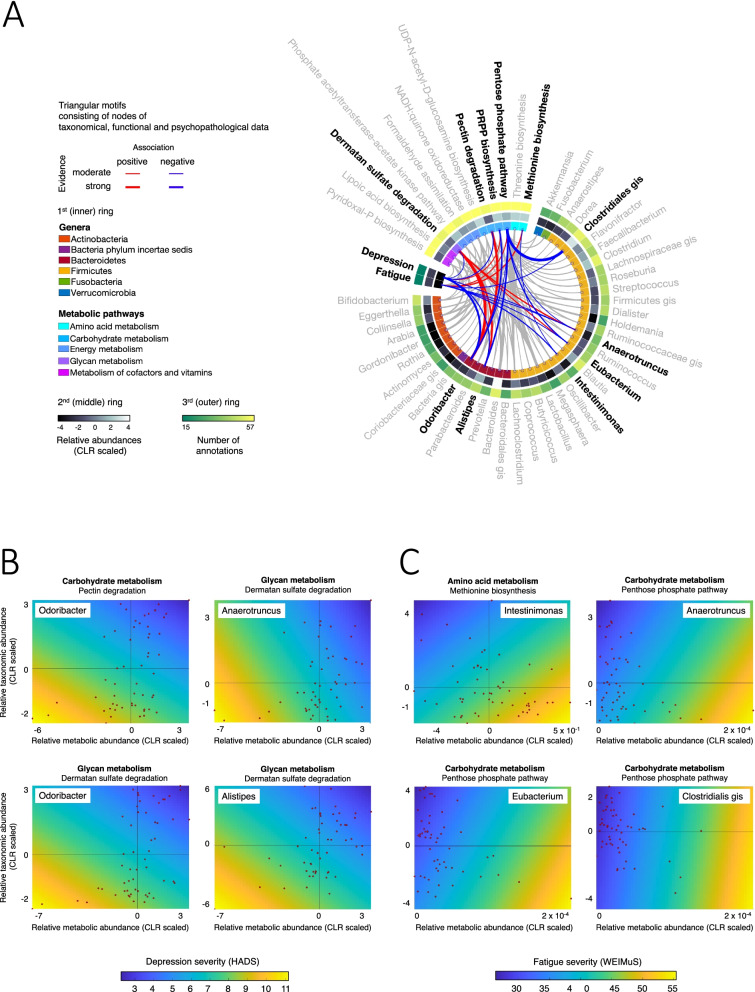
Results of motif analysis of correlation network topology. **A** Ring graph showing significant correlations of gut bacterial genera/metabolic modules with depression and fatigue severity. Triangular motifs associating both a bacterial taxon and a metabolic module with depression and fatigue are coloured (and taxa shown in boldface), and all other associations are displayed in grey. Those belonging to triangular motifs are displayed in red or blue (see colour key). Line thickness is proportional to association strength. On the inner ring, phyla and metabolic pathways are colour coded. On the second ring, relative abundances are coded as a grey-scale heatmap. On the outer ring, the number of patients in which the genus/metabolic module was found is colour coded from green to yellow. **B** Scatter plots showing taxonomic versus metabolic abundances for selected motif triplets (individual data points shown as red dots) with psychopathological severity values colour coded in the background (depression and fatigue severity increases from blue to yellow)

The results are listed in Table 2 and displayed in Fig. 4. Single and double associations are also displayed in these figures (grey).

**Table 2:** Triangular motifs for depression severity and taxonomic and functional abundances. Listed are all triangular motifs within the joint network, formed by taxonomic-metabolic, taxonomic-psychopathological and metabolic-psychopathological associations. Related associations are alternately highlighted in grey or white

Association		Log_**10**_BF(10)	Rho
**Depression**			
Bacteroidetes: *Odoribacter*	Carbohydrate metabolism: pectin degradation^a^	2.026	0.455
Bacteroidetes: *Odoribacter*	Depression: HADS	1.956	− 0.457
Carbohydrate metabolism: pectin degradation^a^	Depression: HADS	0.804	− 0.343
Bacteroidetes: *Odoribacter*	Carbohydrate metabolism: PRPP biosynthesis^b^	1.291	− 0.390
Bacteroidetes: *Odoribacter*	Depression: HADS	1.956	− 0.457
Carbohydrate metabolism: PRPP biosynthesis^b^	Depression: HADS	0.655	0.324
Bacteroidetes: *Odoribacter*	Glycan metabolism: dermatan sulfate degradation^c^	2.611	0.498
Bacteroidetes: *Odoribacter*	Depression: HADS	1.956	− 0.457
Glycan metabolism: dermatan sulfate degradation^c^	Depression: HADS	0.530	− 0.307
Bacteroidetes: *Alistipes*	Glycan metabolism: dermatan sulfate degradation^c^	1.644	0.423
Bacteroidetes: *Alistipes*	Depression: HADS	0.997	− 0.365
Glycan metabolism: dermatan sulfate degradation^c^	Depression: HADS	0.530	− 0.307
Firmicutes: *Anaerotruncus*	Glycan metabolism: dermatan sulfate degradation^c^	1.098	0.370
Firmicutes: *Anaerotruncus*	Depression: HADS	0.530	− 0.307
Glycan metabolism: dermatan sulfate degradation^c^	Depression: HADS	0.666	− 0.326
**Fatigue**			
Firmicutes: *Intestinimonas*	Amino acid metabolism: methionine biosynthesis^d^	0.509	− 0.300
Firmicutes: *Intestinimonas*	Fatigue: WEIMuS	0.972	− 0.363
Amino acid metabolism: methionine biosynthesis^d^	Fatigue: WEIMuS	0.959	0.361
Firmicutes: *Eubacterium*	Carbohydrate metabolism: pentose phosphate pathway^e^	1.082	− 0.369
Firmicutes: *Eubacterium*	Fatigue: WEIMuS	0.655	− 0.324
Carbohydrate metabolism: pentose phosphate pathway^e^	Fatigue: WEIMuS	0.715	0.332
Firmicutes: *Anaerotruncus*	Carbohydrate metabolism: pentose phosphate pathway^e^	0.554	− 0.306
Firmicutes: *Anaerotruncus*	Fatigue: WEIMuS	0.662	− 0.325
Carbohydrate metabolism: pentose phosphate pathway^e^	Fatigue: WEIMuS	0.715	0.332
Firmicutes: *Clostridiales genus incertae sedis*	Carbohydrate metabolism: pentose phosphate pathway^e^	2.900	− 0.517
Firmicutes: *Clostridiales genus incertae sedis*	Fatigue: WEIMuS	0.660	− 0.325
Carbohydrate metabolism: pentose phosphate pathway^e^	Fatigue: WEIMuS	0.715	0.332

^a^KEGG—path: map00040—map01100

^b^KEGG—path: map00030—map00230—map01200—map01230—map01100

^c^KEGG—path: map00531—map01100

^d^KEGG—path: map00270—map01230—map01100

^e^KEGG—path: map00030—map01200—map01230—map01100—map01120

## Discussion

The present study examined the metagenomic microbiota profiles of patients with active IBD and their associations with depression and fatigue severity. It yielded the following major findings.

First, triangular motif analysis identified triple associations between taxonomical and functional faecal microbiota parameters and symptoms of depression and fatigue. Second, neither depression nor fatigue was associated with microbiome alpha diversity or inflammatory activity as determined by serum CRP or faecal calprotectin levels. Third, the taxonomical abundance of several SCFA-producing genera (*Odoribacter*, *Alistipes*, *Anaerotruncus*, *Intestinimonas*, *Eubacterium* and *Clostridiales g.i.s*.) was negatively associated with depression and/or fatigue. Fourth, functional microbiome analysis linked depression to glycan and pectin metabolism as well as PRPP biosynthesis, and fatigue to methionine biosynthesis and the pentose phosphate pathway. Finally, microbiome findings showed partially overlapping but also partially distinct taxonomic and functional associations for depression and fatigue, respectively.

### Depression

Depression is a leading health issue with an increasing prevalence worldwide and a higher prevalence in persons with IBD compared to the general population, especially during active disease [50]. While it is conceivable that a chronic and complex disorder such as IBD may impair quality of life by different mechanisms, such as physical symptoms, stigma and hospitalisations, the relationship between IBD and depressive symptoms appears to go beyond low mood caused by the low quality of life.

In support of bidirectional etiological links between IBD and depression, there is mounting evidence of an increased risk for developing IBD with pre-existing depression [51–53] and of mood disorders preceding the onset of IBD and other immune-mediated disorders for years [53–55]. Numerous studies have investigated the relationship between depressive symptoms and the course of IBD and reported bidirectional associations [56, 57], and there is growing data on the beneficial effect of antidepressants on the course or development of IBD [51, 58–61].

Taken together, increasing evidence supports the theory of biological mechanisms linking gut inflammation and depressive symptoms along the gut-brain axis or microbiota-gut-brain axis [4]. There is, however, still very limited data connecting depression in IBD with the gut microbiome [24, 25]. Neither of the existing studies included functional or metabolic information, and one examined a very limited sample of only 15 patients. Nevertheless, numerous studies have connected depressive syndromes in general with changes in the gut microbiome [16, 17, 19, 62], and preliminary evidence even points to a causal contribution of the gut microbiota as suggested by the transferability of depressive behaviour from patients to mice via faecal microbiota transplant [25, 63].

Antidepressants were shown to influence bacteria or the gut microbiome in vitro and in vivo [64–67]. Vice versa, probiotics can have antidepressant effects [68]. The bidirectional effects of antidepressants on the microbiome and of microbiota manipulation on depression also point to a role of dysbiosis in the development of depression. In conclusion, one might speculate that the efficacy of antidepressants as well as probiotics on depressive symptoms as well as IBD activity may at least in part be mediated by an impact on dysbiosis, and this may explain why some people respond to medication while others do not, depending on the extent to which depressive symptoms in an individual are mediated by dysbiosis. In our study, examining the confounding factors for taxonomic abundance revealed an effect of antidepressant medication on several taxa (see Additional file 2: Fig. S1), which is why we controlled for this factor among others.

Anti-inflammatory medication, e.g. with tumour necrosis factor-alpha inhibitors, such as infliximab, was shown to improve depression in inflammatory disorders [69, 70], which may relate to an improvement of the dysbiotic state of a patient’s microbiome. Of note, a randomised clinical trial examining the effects of infliximab in major depression disorder showed no direct effects on depression scores in the whole infliximab-treated group, but interestingly in a subgroup of patients with adverse childhood events [71], i.e. traumatic experiences during early life. Considering that childhood is a crucial phase for the development of a stable microbiome, it is possible that these effects were also mediated by the changes in the microbiota, rather than by changes in inflammation. In line with these findings, CRP values as a measure of systemic inflammatory activity and faecal calprotectin as a marker of luminal inflammation in the present study did not associate with psychometric scores (see Fig. 3).

Triangle motifs of taxonomic and functional microbiome profiles with depression scores implicated 3 genera and 3 metabolic pathways to be associated with depression. We identified two genera of the phylum Bacteroidetes (*Odoribacter* and *Alistipes*) and one genus of the phylum Firmicutes (*Anaerotruncus*) to be negatively associated with depression scores. Functional modules associated with depression implicate glycan metabolism, which is in line with previous studies [19, 72], along with pectin and other carbohydrate metabolisms.

We found microbial pectin degradation and glycan metabolism to negatively correlate with depression scores and positively associate with the abundance of the 3 implicated genera. Pectins and some glycans are indigestible dietary compounds foraged by gut bacteria, which produce short-chain fatty acids (SCFA) in the degradation process [73]. In line, a reduced abundance of SCFA-producing microbial taxa and pathways has previously been associated with major depression disorder [74]. In a mouse model of depression, reduced SCFA levels were identified after chronic unpredictable mild stress, and intrarectal application of propionate could alleviate depression-like behaviour in these mice [75]. In IBD, the protective anti-inflammatory effects of SCFA have been repeatedly reported. These effects include the inhibition of proinflammatory pathways, such as NFkappaB-activation [76] or the stimulation of regulatory T-cells [77]. As SCFAs can reach systemic circulation and pass the blood-brain barrier, they seem to play an important role in microbiota-brain-gut interactions [78, 79]. Of note, all three genera implicated in triangular motifs in the present study have also previously been shown to produce SCFA [80, 81], emphasising the potential role of this metabolic pathway in the development of depression in IBD.

Furthermore, glycans are an important part of the mucous layer separating the gut microbiome from the intestinal epithelium, and changes in microbial glycan metabolism in this study may also implicate impaired barrier function to contribute to the association between dysbiotic microbiota and systemic pathology including depression [82]. In line, butyrate, which is produced by bacteria such as *Anaerotruncus*, can strengthen intestinal epithelial barrier function [83]. As gut barrier function is certainly affected by intestinal inflammation in active IBD, an increase of circulating pro-inflammatory cytokines due to increased permeability is another possible mechanism of depressive symptoms being mediated by microbiota changes.

Taken together, mounting evidence supports the importance of the microbiome in depression, but a mechanistic understanding is lacking. Our study provides the first evidence of a triangular association between structural and functional microbiota parameters and depression scores, implicating a reduction of SCFA-producing genera and pathways and possibly impaired barrier function to be associated with depression. Future research should specifically address these pathways to increase our understanding of the development of extraintestinal symptoms in IBD.

### Fatigue

Fatigue is a burdensome symptom that leads to a considerably reduced QoL [84] and is reported by 50 to 80% of patients suffering from IBD [1]. Fatigue is a multidimensional problem with different definitions used in different scientific backgrounds. Using the WEIMuS score, we addressed the two main domains of physical and mental fatigue [31], and while there is some association with depression and anxiety, fatigue has to be recognised as an independent symptom [85]. Some therapeutic approaches have been investigated to relieve the patients’ burden, yet these interventions resulted in no or only minimal effect [86] despite covering a wide variety of approaches from electroacupuncture to cognitive behavioural therapy to pharmacological interventions. Therefore, a better understanding of the aetiology of fatigue in IBD patients is needed to provide a more promising approach to therapy. While the presence of fatigue and IBD are clearly connected and the prevalence of fatigue is higher in active disease, the impact of fatigue on the patient’s QoL may be independent of the activity of the IBD [87].

In the present study, neither CRP as a marker of active inflammation nor gut microbial alpha diversity were associated with fatigue symptom severity. This implies that these rather broad markers of inflammation and dysbiosis cannot satisfyingly explain the variance in fatigue or distinguish between patients with and without fatigue in this sample with active inflammation, unlike a previous study reporting such an association in remitted patients [26]. Whether this finding results from a lack of power in our cohort or represents an independent finding has to be determined in a follow-up investigation. The same study also found a reduction in the serum levels of tryptophan, proline, methionine and sarcosine along with a reduction of *F. prausnitzii* and *Roseburia hominis* in the group of fatigued IBD patients. Therefore, we focused on the interaction between composition and function of intestinal microbiota with fatigue for further analysis. In the present study, we associated the changes in psychometry with changes in taxonomic and functional microbial gene abundances. Our analysis showed that higher fatigue scores are associated with a decrease in the abundance of *Intestinimonas* and an increase in the amino acid metabolism pathway. *Intestinimonas* has the unique ability to degrade Amadori products (fructosamines) and especially fructoselysine into butyrate. Amadori products are non-enzymatic reaction products between sugars and free amino groups that are produced when food is heated. By ingesting a typical Western diet, a person can ingest 500–1200 mg of these Amadori products daily [88]. About 70–90% of these are not absorbed in the upper intestine but metabolised by gut bacteria. While *Intestinimonas* produces butyrate [89], fructosamines can be used by other bacteria, e.g. *E. coli* [90] as a source for glucose, leaving lysine and other amino acids as additional products. While there is no proven link between these pathways and fatigue yet, both SCFA and amino acids [91, 92] have neuromodulatory properties and might therefore be relevant in the pathogenesis of fatigue in these patients. Furthermore, our analysis showed a positive correlation of fatigue scores with activation of the pentose phosphate pathway, especially the metabolisation of ribose 5 phosphate to fructose 6 phosphate. While we could not find any data connecting this pathway directly with fatigue, one might speculate that it could be connected to a reduced or modified pool of SCFAs. A conceivable mechanism for this process is that the reduced prevalence of bacteria metabolising C5 and C6 sugars using the sedoheptulose-1,7-bisphosphate pathway [93] results in a reduction in the metabolisation of C5 and C6 sugars derived from plant-based polymers to SCFAs [94] while they get metabolised by transaldolase positive bacteria, using the pentose phosphate pathway, like *Enterococcus faecalis* that do not participate in SCFAs generation. Depletion of SCFA-producing taxa has also been previously reported in myalgic encephalomyelitis/chronic fatigue syndrome [20] and fatigue symptoms in cancer [22], and this reduction in SCFA production might also be one of the components leading to fatigue in our patients.

### Fatigue and depression in IBD

In the investigation of fatigue and depression in IBD, it is noteworthy that these symptoms can be challenging to disentangle. Not only do they show considerable symptom overlap, but they also influence each other. Patients with IBD who suffer from fatigue may have an underlying depression or feel depressed because of the lack of energy. One way to address this issue and attempt to understand shared or possible fatigue- or depression-specific underlying biochemical microbiota-dependent mechanisms is to collect information on both symptoms in the same cohort. Shared microbial associations that were associated with both depression and fatigue scores in our cohort implicate the genus *Anaerotruncus*, a butyrate producer [95]. *Anaerotruncus* species have previously been connected to autoimmune [96] as well as metabolic disorders [97, 98].

As SCFA production was associated with both depression and fatigue in this and other studies, interventional research regarding this relationship in IBD is warranted. Of note, SCFA production is strongly connected to nutrition and especially fibre intake. Studies have examined the relationship between nutrition and depression [99–101] and promoted “anti-inflammatory” microbiota-directed diets that may reduce fatigue [102] and depression [101, 103]. The impact of nutrition on the onset [104] and course of IBD has been under investigation for many years, indicating an impact of nutrition on both intestinal inflammation and associated extraintestinal symptoms. The results of the present study underline the potential of microbiota-directed dietary interventions with the specific aim of reducing fatigue and depression in persons with IBD.

While decreased SCFA metabolism may contribute to both depression and fatigue, functional modules identified by triple association indicate separate pathways by which the microbiome may be contributing to the development of these symptoms, which should be investigated in future research.

### Limitations

We have to acknowledge several limitations of this study. The sample size is limited and heterogeneous with regard to diagnosis, age and (previous) medication due to the exploratory character of this study and the screening of consecutive patients with active disease. The cross-sectional design and the undirected and correlative nature of the analysed joint network do not provide causal information, and our findings remain to be confirmed in longitudinal and interventional studies. Furthermore, the dimensional approach to extraintestinal symptoms in this unselected patient sample (i.e. not selected with regard to depression or fatigue) was not designed to distinguish patients with clinically relevant depression or fatigue from others. However, we believe that a dimensional approach can reduce the risk of selection bias. Also, given the distribution of fatigue and depression scores with many patients scoring close to recommended cut-off scores for relevant symptom load on both sides, a categorical approach might have induced a false separation between the groups and thus led to additional bias. With regard to nuisance variables, we did not control for alcohol consumption, which was shown to influence microbiota composition in an important recent study [42]. Unfortunately, as the mentioned work was published after the recruitment period of our study, alcohol consumption was not assessed in our cohort. It was also unfeasible to control for stool consistency [42], as this information was not provided by all patients. These limitations with respect to unmeasured potential confounders might be a source of bias in our results. Finally, we used short self-reported questionnaires to measure the symptoms of interest, which in the case of depression may not be as reliable as a psychiatric interview. However, the HADS is commonly used in IBD research and was previously compared to structured psychiatric interviews in patients with IBD, where its validity and reliability to detect depressive symptoms were confirmed along with the highest specificity for depression among the examined scales [30]. The chosen instrument to measure fatigue symptoms (WEIMuS) is not an IBD-specific questionnaire. Although it has been used in IBD studies before [32], it has not been formally validated in an IBD cohort. It was primarily chosen because at the time of the study, no German IBD-specific fatigue questionnaire was available, and a questionnaire for multiple sclerosis as another immune-mediated inflammatory disorder appeared more suitable than other available, mostly cancer-related, instruments. Also, the comprehension of cognitive as well as physical fatigue in this questionnaire appeared suitable for our purposes.

## Conclusions

This study provides the first evidence of co-occurring taxonomic and functional microbiota changes associated with symptoms of depression and fatigue in persons with active IBD. Genera and functional pathways associated with depression indicate a role of SCFA-producing taxa as well as glycan and pectin metabolisms. Fatigue was also associated with a decrease in SCFA producers and functional changes in amino acid as well as central carbohydrate metabolism. While fatigue and depression are highly overlapping syndromes and often co-occur in patients with active IBD, triangular motifs implicate shared yet partly separate pathways that may be involved in the development of these extraintestinal symptoms. These findings increase our understanding of extraintestinal symptoms in active IBD with considerable impact on quality of life and open possibilities for future research targeting these symptoms by addressing microbiota-brain-gut interactions in IBD.

## Supplementary Information

**Additional file 1: Table S1.** Associations between clinical characteristics of the study sample and fatigue/depression.

**Additional file 2: Fig. S1.** Associations between demographical (age, sex) and clinical (diagnosis, CRP) variables and medication (steroids, 5-ASA, Immunosuppressants, Hormonal contraception, Antidepressants, PPIs) on taxonomical and metabolic abundances as assessed by linear regression modeling. Displayed are heat maps of port-hoc t-test results for each explanatory variable. Positive t-values displayed in white to red colour indicate a positive association, those displayed in white to blue colours indicate a negative association or negative t-values respectively. Bar graphs right beside the heat maps show the allover variance explanation (*R*^2^) of the linear regression model. All genera and metabolic modules with an at least moderate association level (indicated by the thin grey line in bar plot graphs) are mentioned at the y-axis of the heat map.

## Data Availability

All data needed to evaluate the conclusions in the paper are present in the paper and/or the supplementary material. Raw sequencing data is uploaded and available via the *European Nucleotide Archive* [105]. Parts of the clinical data can be provided by the corresponding author’s pending scientific review and a completed material transfer agreement. Requests for underlying data should be submitted to anne.thomann@medma.uni-heidelberg.de.

## Abbreviations

BF: Bayes factor
CD: Crohn’s disease
CLR: Centred log ratio
CRP: C-reactive protein
g.i.s.: *genus incertae sedis*
HADS: Hospital Anxiety and Depression Scale
IBD: Inflammatory bowel disease
JZS: Jeffreys-Zellner-Siow
KEGG: Kyoto Encyclopedia of Genes and Genomes
KO: KEGG orthology
OTU: Operational taxonomic unit
PCR: Polymerase chain reaction
SCFA: Short-chain fatty acids
UC: Ulcerative colitis
WEIMuS: Wurzburg Fatigue Inventory Multiple Sclerosis
