# Supplementary material for: Depression and fatigue in active IBD from a microbiome perspective—a Bayesian approach to faecal metagenomics
Source: BMC Med. 2022 Oct 17;20:366. doi: 10.1186/s12916-022-02550-7 (PMC9575298; doi:10.1186/s12916-022-02550-7)
Supplement: Supplementary file 1 — Additional file 1: Table S1. Associations between clinical characteristics of the study sample and fatigue/depression. [file 12916_2022_2550_MOESM1_ESM.docx]

**Table S1: Associations between clinical characteristics of the study sample and fatigue/depression**

| **Clinical characteristics** | **n (%)** | **Fatigue (WEIMuS-Scores)** | | | | | | | | | | **Depression (HADS-D-Scores)** | | | | | | | |
| --- | --- | --- | --- | --- | --- | --- | --- | --- | --- | --- | --- | --- | --- | --- | --- | --- | --- | --- | --- |
|  |  | Mean (SD) | n ≥ 32P. (%) | | Correlation | | | | T-Statistics | | | Mean (SD) | n ≥ 10P. (%) | Correlation | | | T-Statistics | | |
|  |  |  |  |  | Log_10_BF10 | | ρ | | Log_10_BF10 | | T |  |  | Log_10_BF10 | | ρ | Log_10_BF10 | | T |
| **Prior bowel resection** | 24 (39%) | 32.2 (15.7) | 12  (50%) | |  | | | | **-0.559** | | 0.278  p = .782 | 6.1 (4.1) | 7 (29%) |  | | | **-0.513** | | 0.581,  p = .563 |
| **No resection** | 38 (61%) | 31.1 (13.7) | 18 (47%) | |  | | | |  |  |  | 6.8 (4.7) | 11 (29%) |  | | |  |  |  |
|  | | | | | | | | | | | | | | | | | | | |
| **Refractory disease** | 22 (35%) | 30.4 (14.1) | 11 (50%) | |  | | | | **-0.530** | | -0.460 p = .647 | 6.8 (4.1) | 5 (23%) |  | | | **-0.545** | | 0.354 p = .725 |
| **No refractory disease** | 40 (65%) | 32.2 (14.8) | 20 (50%) | |  | | | |  |  |  | 6.3 (4.7) | 13 (33%) |  | | |  |  |  |
|  | | | | | | | | | | | | | | | | | | | |
| **Current steroid therapy** | 21 (34%) | 32.3 (14.3) | 12 (57%) | |  | | | | **-0.551** | | 0.301 p = .764 | 5.9 (4.1) | 4 (19%) |  | | | -0.461 | | -0.770 p = .444 |
| **No current steroids** | 41 (66%) | 31.1 (14.7) | 19 (46%) | |  | | | |  |  |  | 6.8 (4.6) | 14 (34%) |  | | |  |  |  |
|  | | | | | | | | | | | | | | | | | | | |
| **Biological therapy** |  |  | | |  | | | |  | | |  | |  | | |  | | |
| **Current** | 10 (16%) | 27.6 (16.7) | 5 (50%) | |  | | | |  | | | 4.9 (2.9) | 1 (10%) |  | | |  | | |
| - TNFi | 3 (4%) | 24.7 | 2 | |  | | | |  | | | 6.7 | 1 |  | | |  | | |
| - VDZ | 5 (8%) | 21.8 | 1 | |  | | | |  | | | 3.2 | 0 |  | | |  | | |
| - UST | 2 (3%) | 46.5 | 2 | |  | | | |  | | | 6.5 | 0 |  | | |  | | |
|  |  | | |  | | | |  | |  | |  | | |  | | |  | |
| **Prior** therapy | 31 (50%) | 28.6 (14.7) | 15 (48%) | |  | | | | -0.112 | | 1.606  p = .114 | 5.9 (4.1) | 6 (19%) |  | | | -0.398 | | 1.003 p = .320 |
| **Naïve** to therapy | 31 (50%) | 34.6 (13.8) | 16 (52%) | |  | | | |  |  |  | 7.1 (4.8) | 12 (39%) |  | | |  |  |  |
|  | | | | | | | | | | | | | | | | | | | |
| **Faecal calprotectin** | 53 (85%) |  | | | **-0.676** | -0.028  p = .706 | | |  | | |  | | **-0.580** | | -0.165 p = .440 |  | | |

**Abbreviations:** BF, Bayes factor; HADS-D, Hospital Anxiety and Depression Scale, depression subscale; SD, standard deviation; TNFi, tumor necrosis factor alpha inhibitor (infliximab or adalimumab); UST, ustekinumab; VDZ, vedolizumab; WEIMuS, Wurzburg Fatigue Inventory Multiple Sclerosis
