# Supplementary figures and images for: Depression and fatigue in active IBD from a microbiome perspective—a Bayesian approach to faecal metagenomics
Source: BMC Med. 2022 Oct 17;20:366. doi: 10.1186/s12916-022-02550-7 (PMC9575298; doi:10.1186/s12916-022-02550-7)

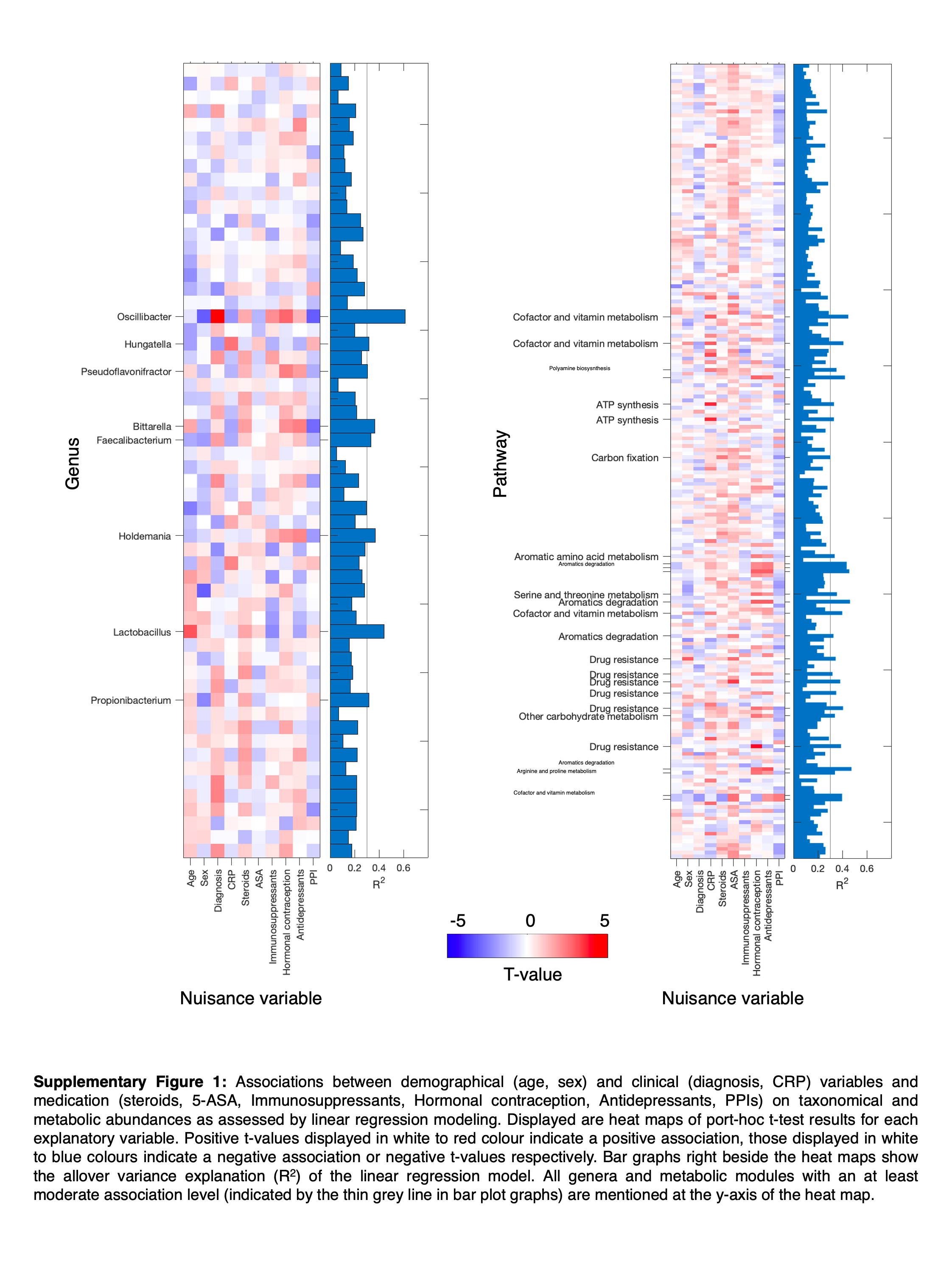

Supplement: Supplementary file 2 — Additional file 2: Fig. S1. Associations between demographical (age, sex) and clinical (diagnosis, CRP) variables and medication (steroids, 5-ASA, Immunosuppressants, Hormonal contraception, Antidepressants, PPIs) on taxonomical and metabolic abundances as assessed by linear regression modeling. Displayed are heat maps of port-hoc t-test results for each explanatory variable. Positive t-values displayed in white to red colour indicate a positive association, those displayed in white to blue colours indicate a negative association or negative t-values respectively. Bar graphs right beside the heat maps show the allover variance explanation (R2) of the linear regression model. All genera and metabolic modules with an at least moderate association level (indicated by the thin grey line in bar plot graphs) are mentioned at the y-axis of the heat map. [file 12916_2022_2550_MOESM2_ESM.tiff]
